# Specific alterations in gut microbiota are associated with prognosis of Budd–Chiari syndrome

**DOI:** 10.18632/oncotarget.23234

**Published:** 2017-12-14

**Authors:** Yu-Ling Sun, Wen-Qi Li, Peng-Xu Ding, Zhi-Wei Wang, Chang-Hua Wei, Xiu-Xian Ma, Rui-Fang Zhang, Yan Wu, Lin Zhou, Ruo-Peng Liang, Yan-Peng Zhang, Yi-Pu Zhao, Rong-Tao Zhu, Jian Li

**Affiliations:** ^1^ Department of Hepatobiliary and Pancreatic Surgery, The First Affiliated Hospital, Zhengzhou University, Zhengzhou, China; ^2^ Institute of Hepatobiliary and Pancreatic Diseases, Zhengzhou University, Zhengzhou, China; ^3^ Department of Radioactive Intervention, The First Affiliated Hospital, Zhengzhou University, Zhengzhou, China; ^4^ Department of Ultrasound Diagnosis, The People’s Affiliated Hospital, Zhengzhou University, Zhengzhou, China; ^5^ Department of Ultrasound Diagnosis, The First Affiliated Hospital, Zhengzhou University, Zhengzhou, China; ^6^ Department of Radiology, The First Affiliated Hospital, Zhengzhou University, Zhengzhou, China; ^7^ Department of Digestive Diseases, The First Affiliated Hospital, Zhengzhou University, Zhengzhou, China

**Keywords:** Budd–Chiari syndrome, gut microbiota, prognosis

## Abstract

Gut microbiota is associated with liver diseases. However, gut microbial characteristics of Budd–Chiari syndrome (B-CS) have not been reported. Here, by MiSeq sequencing, gut microbial alterations were characterized among 37 health controls, 20 liver cirrhosis (LC) patients, 31 initial B-CS patients (B-CS group), 33 stability patients after BCS treatment (stability group) and 23 recurrent patients after BCS treatment (recurrence group). Gut microbial diversity was increased in B-CS versus LC. Bacterial community of B-CS clustered with controls but separated from LC. Operational taxonomic units (OTUs) 421, 502 (Clostridium IV) and 141 (Megasphaera) were unique to B-CS. Genera Escherichia/Shigella and Clostridium XI were decreased in B-CS versus controls. Moreover, nine genera, mainly including Bacteroides and Megamonas, were enriched in B-CS versus LC. Notably, Megamonas could distinguish B-CS from LC with areas under the curve (AUCs) of 0.7904. Microbial function prediction revealed that L-amino acid transport system activity was decreased in B-CS versus both LC and controls. Furthermore, OTUs 27 (Clostridium XI), 137 (Clostridium XIVb) and 40 (Bacteroides) were associated with B-CS stability. Importantly, genus Clostridium XI was enriched in stability group versus both recurrence group and B-CS group. Also, PRPP glutamine biosynthesis was reduced in stability group versus recurrence group, but was enriched in stability group versus B-CS group. In conclusion, specific microbial alterations associated with diagnosis and prognosis were detected in B-CS patients. Correction of gut microbial alterations may be a potential strategy for B-CS prevention and treatment.

## INTRODUCTION

Budd–Chiari syndrome (B-CS) is characterized by hepatic venous outflow tract obstruction at various levels from the small hepatic veins to the inferior vena cava (IVC), resulting from thrombosis or its fibrous sequelae [1, 2]. Lack of blood drainage from the liver leads to portal and/or IVC hypertension, causing hepatocyte necrosis, fibrosis, cirrhosis, acute liver failure and even hepatocellular carcinoma (HCC) [1]. Increasing evidence indicates that there are distinct variations in the B-CS prevalence, aetiological distribution, clinical characteristics, and occlusion sites between Asian and western countries, necessitating the use of different treatment modalities [3, 4]. In Western countries, B-CS is mainly caused by blood system disorders, and patients present with hepatic vein thrombosis [2, 5, 6]. In contrast, IVC obstruction is more common in Asian B-CS patients [7]. However, few risk factors for this disease have been identified, and the pathogenesis remains unclear.

As a symbiotic micro-ecosystem within the body [8, 9], the human intestinal microbiota plays a crucial role in human health and diseases. It is composed of 10^13^ to 10^14^ microorganisms that collectively possess at least 100 times as many genes as the human genome [10]. The gut microbiota is closely associated with various chronic diseases, such as type 2 diabetes [11], obesity [12], non-alcoholic fatty liver disease [13, 14], chronic liver injury [15], liver cirrhosis (LC) [16], and HCC [17], in addition to liver transplantation [18]. Liver disease develops following alterations in intestinal permeability and gut microbiota [19], and gut microbial dysbiosis in turn promotes liver disease progression [20]. Notably, hepatic disease improvement may promote gut microbial restoration, which may further benefit the liver through positive feedback of the “gut-liver axis.” [15]

Gut microbial dysbiosis is unique for each chronic disease. In LC, potentially pathogenic bacteria, including *Enterobacteriaceae*, *Veillonellaceae* and *Streptococcaceae*, are prevalent, along with reductions in beneficial populations, such as *Lachnospiraceae* [16]. Analysis of gut microbial alterations in 98 LC patients and 83 healthy controls based on only 15 microbial biomarkers has resulted in establishment of an accurate patient discrimination index [21]. Long-term lack of blood drainage from the liver results in hepatocyte fibrosis and cirrhosis [1]; thus, B-CS always develops after LC. Therefore, we speculate that there is a close relationship between the gut microbiota and B-CS.

However, the gut microbial characteristics of B-CS patients have not yet been reported. This study prospectively collected 144 human stool samples from 37 healthy controls, 20 LC patients and 31 B-CS patients, as well as 33 stable patients after B-CS treatment and 23 recurrent patients after B-CS treatment, for characterization of the B-CS gut microbiota based on 16S rDNA analysis by MiSeq sequencing. The 33 stable patients and 23 recurrent patients were also used to identify the unique bacterial composition associated with B-CS recurrence.

## RESULTS

After applying stringent inclusion and exclusion criteria, 144 subjects were included (Table 1, Supplementary Data File 1) consisting of 37 healthy controls, 20 LC patients, 31 B-CS patients, 33 stable patients and 23 recurrent patients. No significant differences in age, gender or BMI were observed among these groups. Serum alanine aminotransferase, aspartate aminotransferase and glutamyl transpeptidase levels were significantly increased in LC group and B-CS group versus healthy controls; however, no obvious differences were detected between LC group and B-CS group. Albumin level was decreased and direct bilirubin was increased in LC group versus B-CS group, but Child-Pugh score did not significantly differ (*P* = 0.382).

**Table 1:** Clinical characteristics of the enrolled participants

Clinical and pathological indexes			Healthy controls (*n* = 37)	Liver cirrhosis (*n* = 20)	B-CS (*n* = 31)	*P* value ((LC vs. B-CS)	Stability (*n* = 33)	Recurrence (*n* = 23)	*P* value (Sta vs. Rec)
Age (year)			48.1 ± 12.2	47.6 ± 7.2	46.1 ± 8.6	0.512	44.9 ± 8.1	48.9 ± 11.5	0.130
Gender		Female	10.0 (27%)	7.0 (35%)	8.0 (25.8%)	0.482	11.0 (33.3%)	7.0 (30.4%)	0.819
		Male	27.0 (73%)	13.0 (65%)	23.0 (74.2%)		22.0 (66.6%)	16.0 (69.6%)	
BMI			23.07 ± 2.27	24.45 ± 2.54	25.33 ± 3.26	0.311	24.29 ± 3.55	23.30 ± 3.08	0.284
Mild complications	Yes		0 (0%)	4.0 (20%)	6.0 (19.4%)	1.00	0 (0%)	0 (0%)	–
	No		37 (100%)	16.0 (80%)	25.0 (80.6%)		33.0 (100%)	23.0 (100%)	
ALT (U/L)			20.54 ± 7.37	83.50 ± 20.64	81.29 ± 14.92	0.668	29.75 ± 6.85	29.61 ± 6.83	0.936
AST (U/L)			21.38 ± 4.91	74.95 ± 27.45	78.81 ± 14.58	0.568	28.12 ± 5.09	28.17 ± 4.66	0.969
GGT (U/L)			17.0 (14.0, 28.0)	58.5 (25.0, 109.5)	44.0 (37.0, 62.5)	0.589	20.0 (18.0, 31.0)	21.0 (19.0, 33.5)	0.665
Total protein (g/L)			74.87 ± 3.36	60.77 ± 9.58	60.09 ± 7.42	0.779	74.42 ± 3.54	73.87 ± 3.70	0.584
Albumin (g/L)			49.06 ± 2.18	30.22 ± 5.28	36.42 ± 5.81	0.000	49.02 ± 2.26	48.87 ± 1.97	0.792
Total bilirubin (μmol/L)			13.19 ± 4.61	32.92 ± 24.36	24.56 ± 17.6	0.162	13.2 ± 3.28	13.91 ± 4.32	0.552
Direct bilirubin (μmol/L)			4.46 ± 1.50	21.46 ± 17.34	13.67 ± 10.02	0.080	4.79 ± 1.75	4.22 ± 1.28	0.178
Prothrombin time (s)			ND	15.15 ± 4.83	13.36 ± 4.11	0.165	12.02 ± 1.372	11.47 ± 1.71	0.192
Platelets (10E9/L)			222.0 (203, 257)	62.5 (45.5, 74.0)	75 (54.5, 116.5)	0.111	75.0 (58.0, 100.0)	75 (51.5, 106)	0.993
Child-Pugh	A		37.0 (100%)	16.0 (80.0%)	25.0 (80.6%)	0.382	33.0 (100%)	23.0 (100%)	–
	B		0 (0%)	4.0 (20.0%)	6.0 (19.4%)		0 (0%)	0 (0%)	
Etiological factors			No	HBV	ND		ND	ND	

Categorical variables are expressed as group percentages and were compared among samples using Pearson’s χ^2^ or Fisher’s exact test. Continuous data are presented as either the mean ± standard deviation (SD) for normally distributed variables or the median (interquartile range) for non-normally distributed variables. Independent sample analysis of variance was used for comparisons of parametric data, whereas the Mann-Whitney *U*-test was used for comparisons of nonparametric data. All of the these statistical tests were 2-sided, with a significance level of <0.05. Statistical analyses were conducted using SPSS version 19.0 for Windows (SPSS Inc., Chicago, IL, USA).

Abbreviations: B-CS, Budd–Chiari syndrome; LC, Liver cirrhosis; Sta, Stability; Rec, Recurrence; BMI, body mass index; ALT, alanine aminotransferase; AST, aspartate aminotransferase; GGT, glutamyl transpeptidase; ND, no detection.

All samples from the 5 groups were pooled into 10 libraries. Further, 4,303,555 qualified reads were filtered from 7,191,376 raw reads for downstream analysis. A total of 10,000 reads were randomly chosen for each sample. Four samples without reads were discarded (overlapping reads with consecutive barcodes were not detected). A total of 98.23% of all qualified reads were clustered into qualified operational taxonomic units (OTUs) generated with randomly chosen qualified reads. Taxonomic annotations were performed for all qualified OTUs (Supplementary Data File 2), and 127 OTUs were discarded owing to a low distribution among all samples. Finally, 560 qualified OTUs were clustered for downstream analysis (Supplementary Data File 3).

### Significant gut microbiota restoration in B-CS patients compared with LC patients

**Figure 1:**
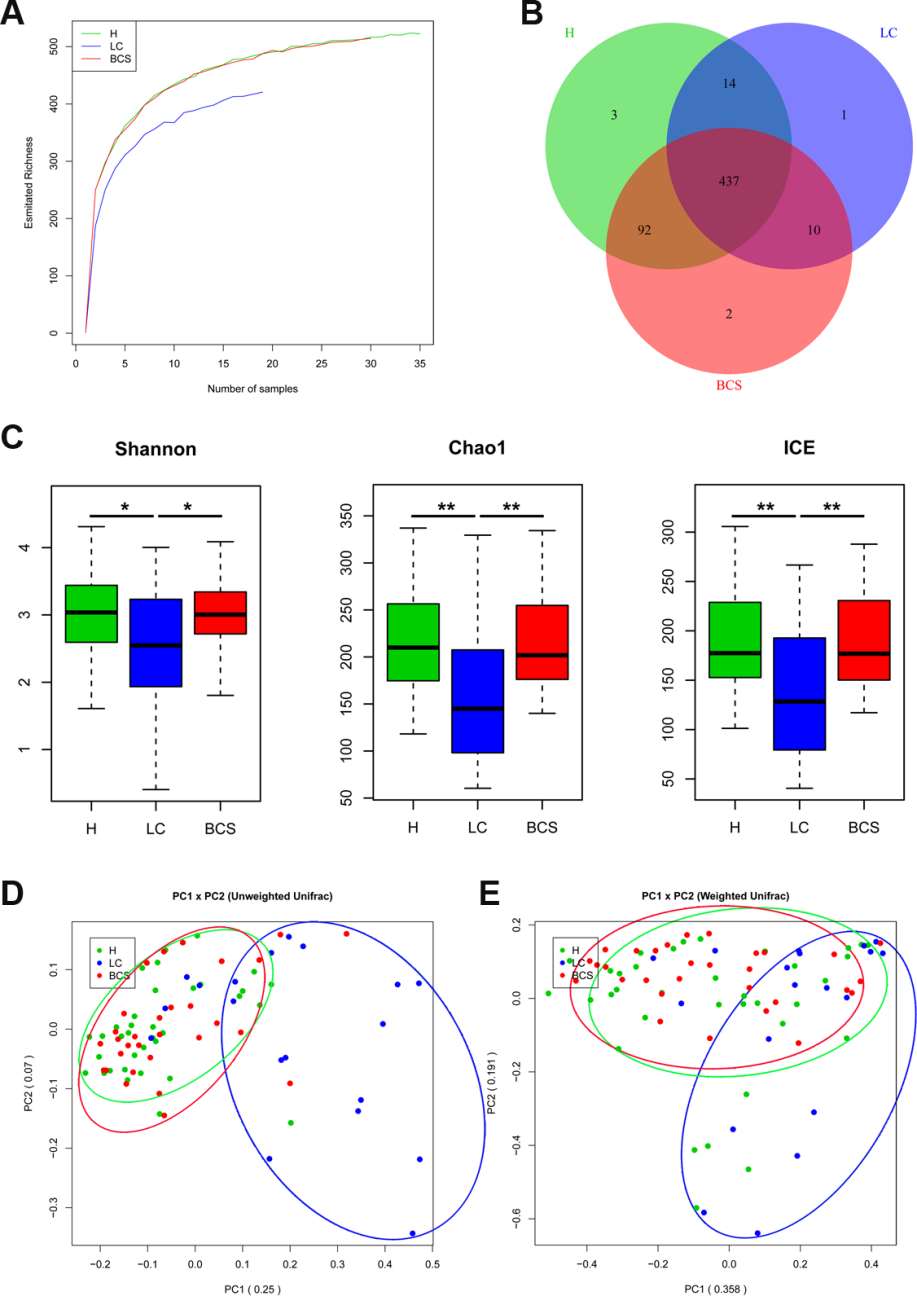
Gut microbial diversity and principal component analysis (PCA) among H, LC patients and B-CS patients (**A**) The relationships between sample number and estimated microbial richness were analysed among healthy controls, LC patients, and BCS patients. (**B**) The Venn diagram presents overlaps among the healthy controls, LC patients, and B-CS patients, as well as the unique OTUs for each group. (**C**) Gut microbial diversity was analysed among the healthy controls, LC patients and B-CS patients by determination of Shannon’s index, the Chao 1 index and incidence-based Coverage Estimators (ICEs). (**D**) PCA based on the unweighted UniFrac metrics of faecal microbiota among the three groups; PC1: 0.25, and PC2: 0.07. (**E**) PCA based on the weighted UniFrac metrics of faecal microbiota among the three groups; PC1: 0.358, and PC2: 0.191. The box presents the 95% confidence intervals (CIs), and the line inside of the box denotes the mean. ^*^P < 0.05, ^**^P < 0.01. H, healthy controls; LC, liver cirrhosis; B-CS, Budd–Chiari syndrome.

To determine the sequencing depths of the gene data sets, the relationship between sample number and estimated richness was analysed. The curves for the three groups tended to be flat (Figure 1A), suggesting that the sequencing data sets were sufficiently large to encompass most of the microbial information. To better understand the shared richness among the three groups, a Venn diagram presenting overlaps among the groups was constructed. Analysis showed that 437 of 560 OTUs were shared among all groups, whereas 447 of 557 OTUs were shared between LC group and B-CS group (Figure 1B).

Gut microbial diversity and species richness were analysed (Supplementary Data File 4). Gut microbial diversity was significantly decreased in LC patients compared with healthy controls (*P* < 0.05 and *P* < 0.01, respectively), while it was markedly increased in B-CS patients compared with LC patients (*P* < 0.05 and *P* < 0.01, respectively), as estimated according to Shannon’s indexes (Figure 1C). Gut microbiota species richness was also similar among the three groups, as shown by the Chao 1 indexes and incidence-based Coverage Estimators (ICEs) (Figure 1C).

To assess similarity among microbial communities, beta diversity was calculated using unweighted and weighted UniFrac distances, and principal component analysis (PCA) was performed. Despite significant inter-individual variation, the unweighted UniFrac plot revealed that bacterial community of LC patients was significantly separated from healthy controls, whereas B-CS patients was clustered with healthy controls but clearly separated from LC patients for principal component (PC) 1 and PC2 (25.0% and 7.0% of explained variance, *P* < 0.001) (Figure 1D). Notably, the weighted UniFrac plot showed similar results for microbial community for PC1 and PC2 (35.8% and 19.1% of explained variance, *P* < 0.001) (Figure 1E). These data suggested that gut microbiota of LC patients showed significant dysbiosis, whereas B-CS patients was obviously restored.

To further compare bacterial community distributions among three groups, another 3 distance analyses (Hellinger distance, Jensen-Shannon Divergence (JSD) analysis, and Spearman distance) were performed, revealing that LC patients was clearly separated from healthy controls, whereas B-CS patients showed obvious restoration versus LC patients (Supplementary Figure 1).

### Unique gut microbiota OTU distribution in B-CS patients

**Figure 2:**
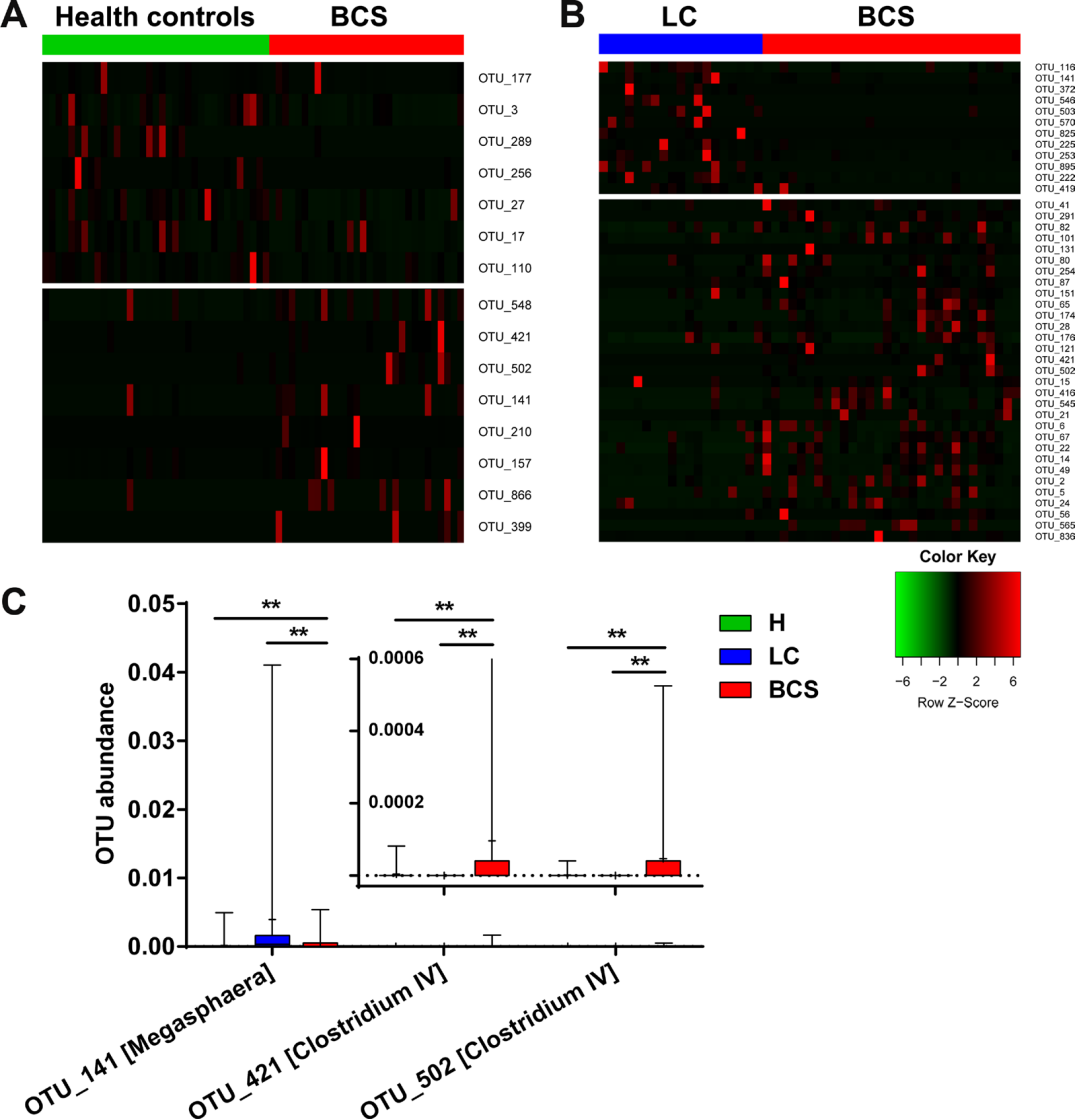
Unique gut microbiota OTU distribution in B-CS patients (**A**) The bundance distribution of 15 OTUs identified as key phylotypes are presented between healthy controls and B-CS patients. (**B**) A total of 43 OTUs exhibited significant differences between B-CS and LC patients. To show the distributions of the OTUs with lower bundances, the coloured squares in each row are scaled to indicate the relative ratio of each OTU among all individuals. (**C**) The shared OTUs with significant differences between the B-CS patients and healthy controls and between the B-CS and LC patients were selected.

To identify key OTU phylotypes in B-CS patients, abundances and distributions of all OTUs were analysed using Wilcoxon rank-sum test. Fifteen OTUs exhibited significant differences between B-CS patients and healthy controls (7 decreased and 8 enriched, Figure 2A). Moreover, we analysed OTU abundance between LC and B-CS patients. Forty-three OTUs displayed significant differences between B-CS and LC patients (12 decreased and 31 enriched, Figure 2B).

To further search for microbial OTUs associated with B-CS, we selected shared OTUs that exhibited significant differences between B-CS and healthy controls, as well as between B-CS and LC patients. The abundances of OTUs 421 and 502, corresponding to *Clostridium IV*, were significantly enriched in B-CS patients versus healthy controls and LC patients (all *P* < 0.01). Additionally, OTU 141, corresponding to *Megasphaera*, was markedly increased in B-CS patients versus healthy controls (*P* < 0.01), but significantly decreased in B-CS versus LC patients (*P* < 0.01) (Figure 2C).

### Phylogenetic profiles of gut microbiota in B-CS patients

**Figure 3:**
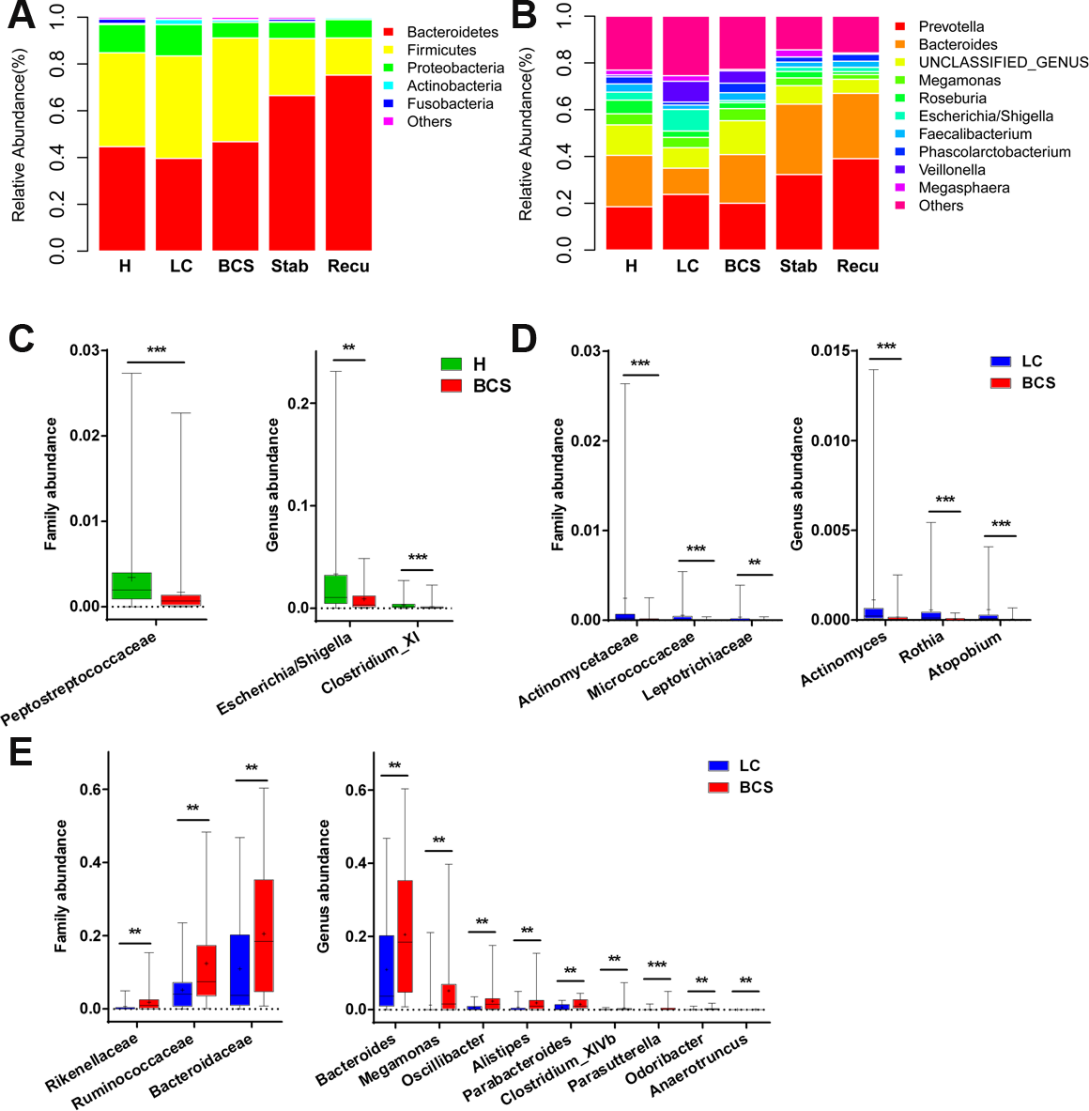
Faecal microbiota compositions among the five groups, as well as the differences in faecal bacterial communities of B-CS patients versus healthy controls and LC patients (**A**) Faecal microbiota composition at the phylum level among the five groups. (**B**) Faecal microbiota composition at the genus level among the five groups. (**C**) Differences in faecal bacterial communities between healthy controls and B-CS patients. (**D**) The bacteria with decreased abundances at the family and genus levels in B-CS versus LC patients. (**E**) The bacteria with increased abundances at the family and genus levels in B-CS versus LC patients. The box presents the 95% confidence intervals, the line inside of the box denotes the median, and the symbol “+” denotes the mean value. ^**^P < 0.01, ^***^P < 0.001.

We further analysed bacterial taxonomic compositions of the five groups. First, we analysed the compositions at the phylum level. Bacterial abundances and compositions of top 5 phyla in each sample among five groups are shown in Supplementary Figure 2A, and the mean abundances in each group are displayed in Figure 3A. *Bacteroidetes*, *Firmicutes* and *Proteobacteria*, collectively representing over 95% of sequences on average, were the three predominant phyla among the five groups. Further, abundances and compositions of top 10 genera in each sample are shown in Supplementary Figure 2B, and mean abundances of top 10 genera in each group are displayed in Figure 3B.

The *Peptostreptococcaceae* family was significantly decreased in B-CS patients versus healthy controls (*P* < 0.001), and at genus level, *Escherichia/Shigella* and *Clostridium XI* were markedly decreased (both *P* < 0.01) (Figure 3C). Moreover, at family level, *Actinomycetaceae*, *Micrococcaceae* and *Leptotrichiaceae* were significantly decreased in B-CS versus LC patients (all *P* < 0.01), whereas *Bacteroidaceae*, *Ruminococcaceae* and *Rikenellaceae* were markedly increased (all *P* < 0.01) (Figure 3D and 3E). Correspondingly, at genus level, *Actinomyces*, *Rothia* and *Atopobium* were obviously decreased (all *P* < 0.001), whereas nine genera, mainly including *Bacteroides*, *Megamonas*, *Oscillibacter*, *Alistipes* and *Parabacteroides,* were substantially enriched in B-CS versus LC patients (all *P* < 0.01) (Figure 3D and 3E).

### Specific bacteria associated with B-CS

**Figure 4:**
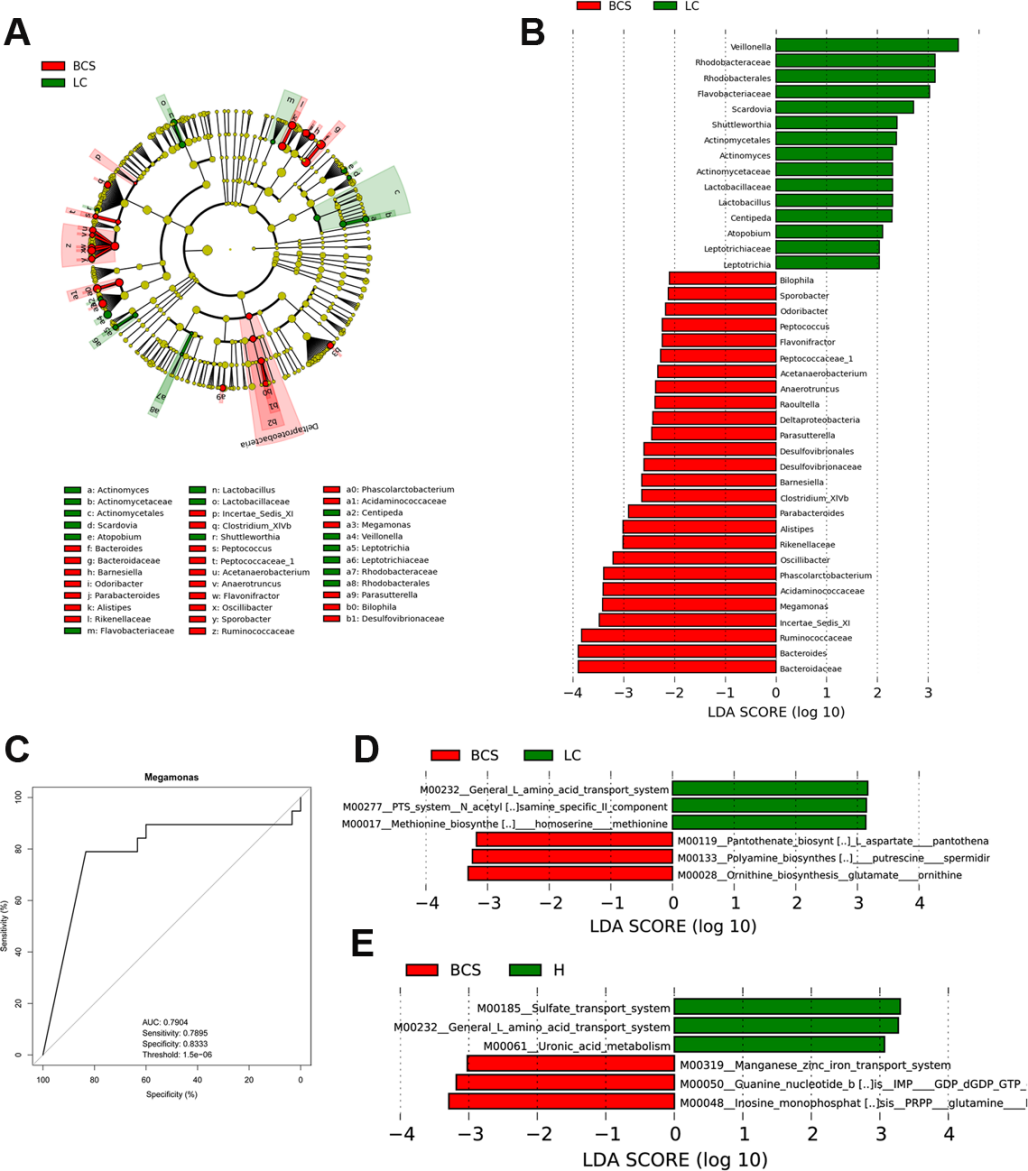
Identification of specific bacterial taxa and microbial functions associated with B-CS (**A**) Phylogenetic profiles of specific bacterial taxa and predominant bacteria in B-CS versus LC patients, as determined using the LEfSe method. (**B**) The greatest differences in taxa between B-CS and LC patients are presented according to the LDA scores (log_10_). (**C**) Receiver operating characteristic (ROC) curve of Megamonas abundance between B-CS and LC patients. (**D**) Differences in gut microbial functions between BCS and LC patients based on the LDA scores (log_10_). (**E**) Differences in gut microbial functions between B-CS patients and healthy controls based on the LDA scores (log_10_). AUC: area under the curve; CI: confidence interval; LEfSe: linear discriminant analysis (LDA) effect size.

To identify bacterial taxa associated with B-CS, we compared gut microbiotas among the groups using the linear discriminant analysis (LDA) Effective Size (LEfSe) method. A cladogram comparing microbial structure and predominant bacteria between LC and B-CS groups is shown in Figure 4A; the greatest differences in taxa between the groups were identified according to the LDA scores (log_10_), as shown in Figure 4B. These data revealed significant differences between both groups. We also detected the greatest differences in taxa between B-CS and healthy controls using the LEfSe method and LDA scores, as shown in Supplementary Figure 3.

We further screened the unique genera as potential biomarkers for distinguishing B-CS from LC patients. Results indicated that *Megamonas* not only was significantly enriched in B-CS patients but also could be used to distinguish B-CS from LC patients, with a high discriminatory power and an AUC of 0.7904 (sensitivity: 0.7895; specificity: 0.8333) (Figure 4C).

Gut microbial phylogeny is closely associated with microbial gene functions; thus, we employed phylogenetic investigation of communities by reconstruction of unobserved states (PICRUSt) version 1.0.0 [22] and human version 0.99 [23] to predict microbial functions using 16S rDNA gene sequences. LDA selection revealed that the pathways L-amino acid transport system, N-acetylgalactosamine PTS system and methionine biosynthesis functions were less abundant, whereas pantothenate biosynthesis, arginine and spermidine biosynthesis, and glutamate ornithine biosynthesis were significantly enriched, in B-CS patients versus LC patients (Figure 4D, Supplementary Data File 5). Results indicated that sulphate transport system, L-amino acid transport system and uronic acid metabolism were less abundant, whereas guanine nucleotide biosynthesis, the manganese/zinc/iron transport system and inosine monophosphate biosynthesis were significantly enriched, in B-CS patients versus healthy controls (Figure 4E, Supplementary Data File 6). Notably, L-amino acid transport system was markedly less abundant in B-CS patients versus both LC patients and healthy controls, demonstrating a unique feature of B-CS.

### Association of gut microbial distribution with B-CS patient prognosis

To further analyse the relationship between the gut microbiota and B-CS prognosis, we assessed 31 B-CS patients, 33 stable patients and 23 recurrent patients.

**Figure 5:**
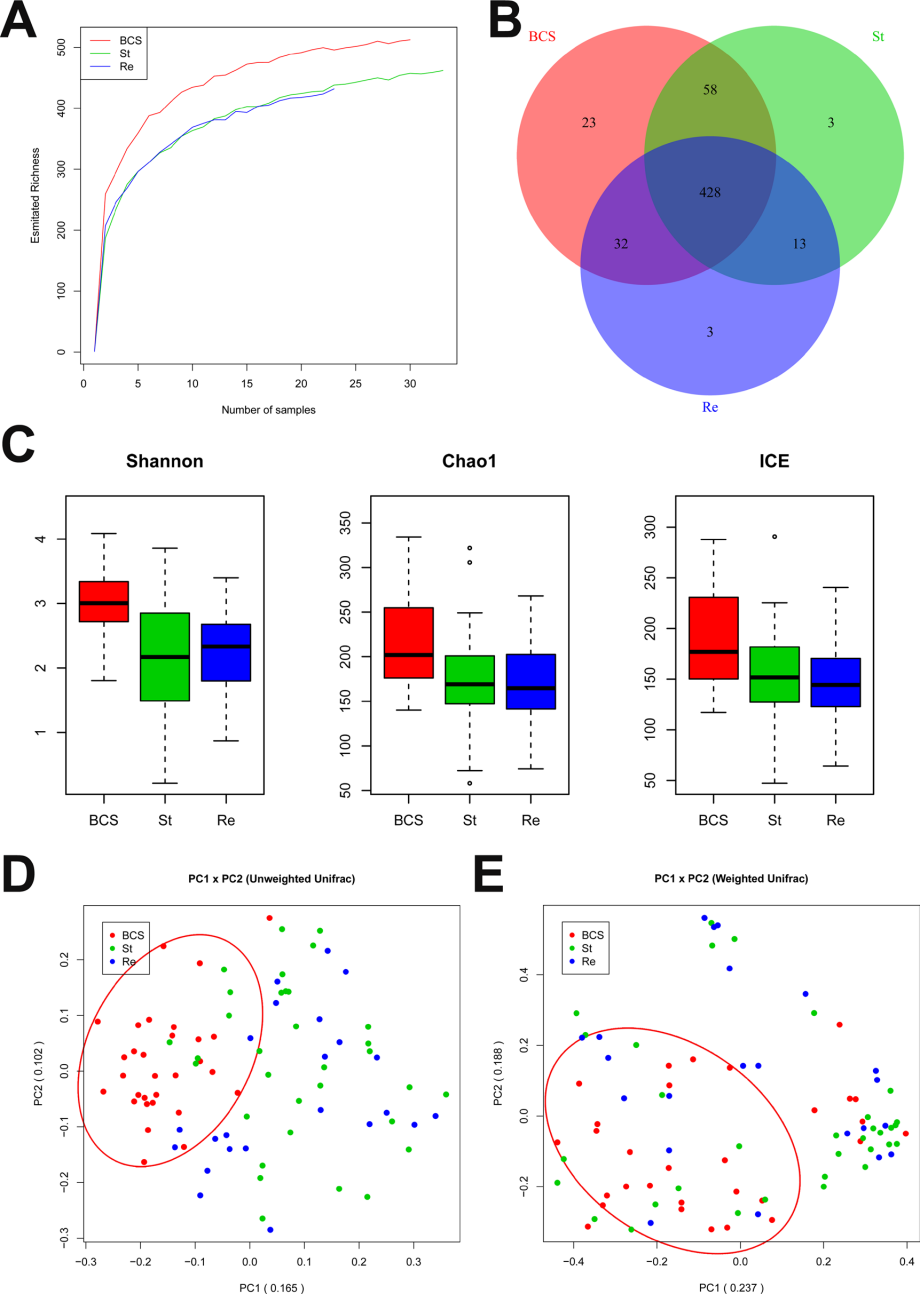
Gut microbial diversity and PCA among B-CS group, stability and recurrence (**A**) The relationships between sample number and estimated microbial richness were analysed among B-CS group, Stability and Recurrence. (**B**) The Venn diagram shows overlaps among B-CS group, Stability and Recurrence, as well as unique OTUs for each group. (**C**) Gut microbial diversity was analysed among B-CS. group, Stability and Recurrence by determining Shannon’s index, the Chao 1 index and ICEs. (**D**) PCA based on the unweighted UniFrac metrics of faecal microbiota among the three groups; PC1: 0.165, and PC2: 0.102. (**E**) PCA based on the weighted UniFrac metrics of faecal microbiota among the three groups; PC1: 0.237, and PC2: 0.188. The box presents the 95% confidence intervals (CIs), and the line inside of the box denotes the mean value. B-CS, Budd–Chiari syndrome; Stability, stable patients; Recurrence, recurrent patients.

First, we analysed the relationship between sample number and estimated richness among the B-CS patients, stable patients and recurrent patients. The curves suggested that the sequencing data were sufficiently large to encompass most of the microbial information (Figure 5A). The Venn diagram showed overlaps among the groups, revealing that 428 of 560 OTUs were shared by all samples, whereas 486 of 557 OTUs were shared between B-CS patients and stable patients, and 441 of 537 OTUs were shared between stable patients and recurrent patients (Figure 5B).

The gut microbial diversity indexes were significantly decreased in both stable patients and recurrent patients compared with B-CS group (both *P* < 0.001), whereas microbial diversity did not obviously differ between stable patients and recurrent patients, as estimated by Shannon’s indexes (Figure 5C). Additionally, gut microbiota species richness was similar among the three groups, as indicated by the Chao 1 indexes and ICEs (Figure 5C).

PCA was conducted based on unweighted and weighted UniFrac distances to assess microbial distribution among the three groups. The unweighted UniFrac plot showed that the gut microbial community of B-CS group was highly separated from those of stable group and recurrent group, whereas no significant separation was observed between stable group and recurrent group, for PC1 and PC2 (16.5% and 10.2% of variance, respectively, *P* < 0.001) (Figure 5D). The weighted UniFrac plot revealed similar results for PC1 and PC2 (23.7% and 18.8% of variance, respectively) (Figure 5E). Moreover, Hellinger, JSD and Spearman analyses showed similar results for gut microbial alterations among the three groups (Supplementary Figure 4). These data suggested that the gut microbiotas of stable group and recurrent group had similar bacterial composition but were significantly separated from that of B-CS group.

### Association of specific gut microbiota OTU distribution with B-CS recurrence

**Figure 6:**
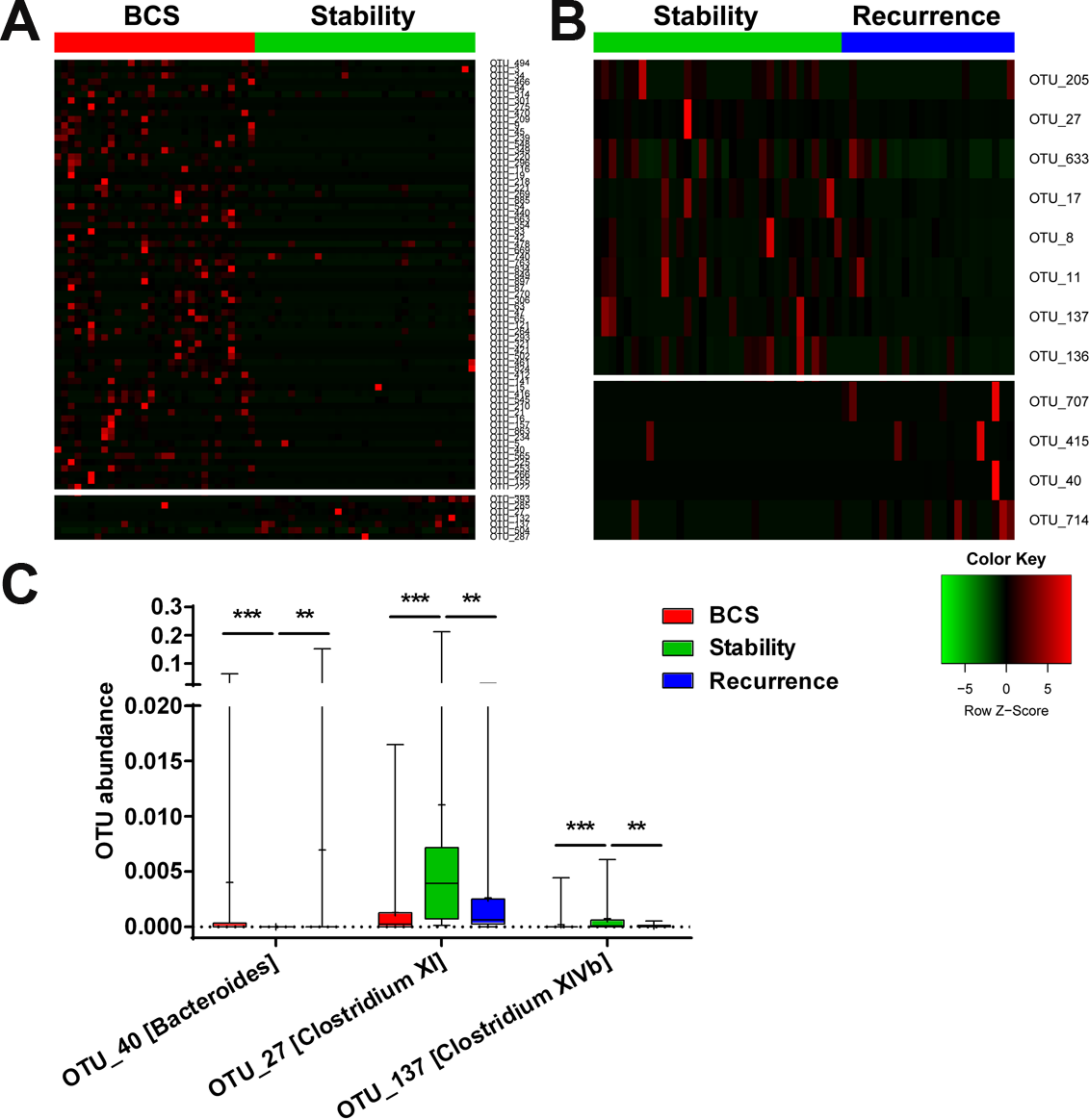
Specific gut microbiota OTU distribution associated with B-CS stability (**A**) Abundance distribution of 76 OTUs identified as key phylotypes between B-CS and stable patients is shown. (**B**) A total of 12 OTUs exhibited significant differences between Stability and Recurrence . To show the distribution of the OTUs with lower abundances, the coloured squares in each row are scaled to depict the relative ratio of each OTU among all individuals. (**C**) The shared OTUs showed significant differences. between B-CS group and Stability, as well as between Stability and Recurrence.

To identify OTUs associated with B-CS recurrence, we compared OTU abundance between B-CS patients and stable patients, as well as between stable patients and recurrent patients. Seventy-six OTUs showed significant differences between stable group and B-CS group (69 decreased and 7 increased) (Figure 6A). Moreover, 12 OTUs exhibited significant differences between stable group and recurrent group (8 increased and 4 decreased) (Figure 6B).

To further assess the OTUs associated with B-CS recurrence, shared OTUs showing significant differences between B-CS patients and stable patients and between stable patients and recurrent group were selected. The abundances of OTUs 27 and 137, corresponding to *Clostridium XI* and *Clostridium XIVb*, were significantly enriched in stable group compared with B-CS and recurrent groups (all *P* < 0.01). In contrast, the abundance of OTU 40, corresponding to *Bacteroides*, was dramatically decreased in stable group versus B-CS and recurrent groups (both *P* < 0.01) (Figure 6C). These data suggested that *Clostridium XI*, *Clostridium XIVb* and *Bacteroides* might be closely associated with B-CS stability.

### Association of gut microbiota taxonomic alterations with B-CS stability

To assess bacterial alterations associated with B-CS stability, we detected bacterial differences between stable group and recurrent group as well as between stable group and B-CS group.

**Figure 7:**
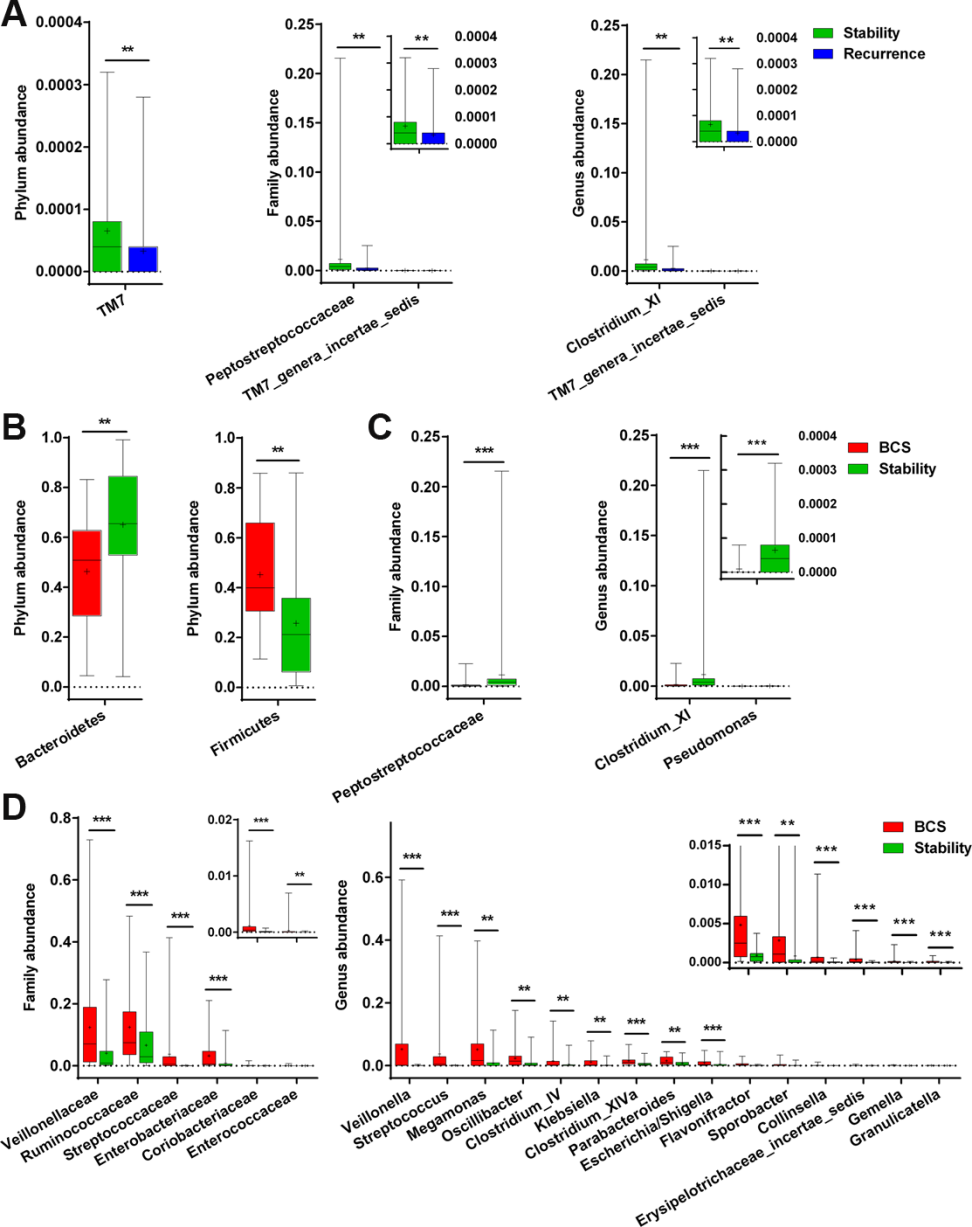
Differences in facial microbiota composition between stable and recurrent groups, as well as between stability and B-CS groups (**A**) Bacterial differences in faecal microbiota between Stability and Recurrence at the phylum, family and genus levels. (**B**) Bacterial differences in faecal microbiota between Stability and B-CS groups at the phylum level. (**C** and **D**) Bacterial differences in faecal microbiota between. Stability and B-CS groups at the family and genus levels. The box presents the 95% confidence intervals, the line inside of the box denotes the median, and the symbol “+” indicates the mean value. ^**^P < 0.01, ^***^P < 0.001.

The phylum TM7 was significantly enriched in stable group compared with recurrent group (*P* < 0.001), and the *Peptostreptococcaceae* family and *incertae sedis* TM7 genera were also increased (both *P* < 0.01). At the genus level, *Clostridium XI* and *incertae sedis* TM7 genera were markedly increased in stable group (both *P* < 0.01) (Figure 7A).

Moreover, the phyla *Bacteroidetes* and *Firmicutes* were significantly increased and decreased, respectively, in stable group compared with B-CS group (both *P* < 0.01) (Figure 7B). Correspondingly, the family *Peptostreptococcaceae* was increased, whereas 6 families, mainly including *Veillonellaceae*, *Ruminococcaceae* and *Streptococcaceae,* were decreased in stable group (all *P* < 0.001) (Figure 7C and 7D). Further, at the genus level, *Clostridium XI* and *Pseudomonas* were markedly increased, whereas 15 genera, mainly including *Veillonella*, *Streptococcus* and *Megamonas,* were decreased in stable group (all *P* < 0.01) (Figure 7C and 7D). Notably, the genus *Clostridium XI* was significantly enriched in stable group compared with recurrent group and B-CS group (both *P* < 0.01), consistent with the specific OTU distributions.

### Functional prediction of gut microbiota associated with B-CS stability

To identify bacterial taxa associated with B-CS stability, we constructed a representative cladogram comparing gut microbial composition and predominant bacteria between stable group and B-CS group using the LEfSe method (Supplementary Figure 5A) and determined the greatest differences in taxa between the groups according to the LDA scores (log_10_) (Supplementary Figure 5B). The data revealed significant differences in the gut microbiota between the two groups. We also constructed a representative cladogram comparing microbial composition between stable group and recurrent group and detected the greatest differences in taxa using the LEfSe method and LDA scores (Supplementary Figure 5C and 5D).

**Figure 8:**
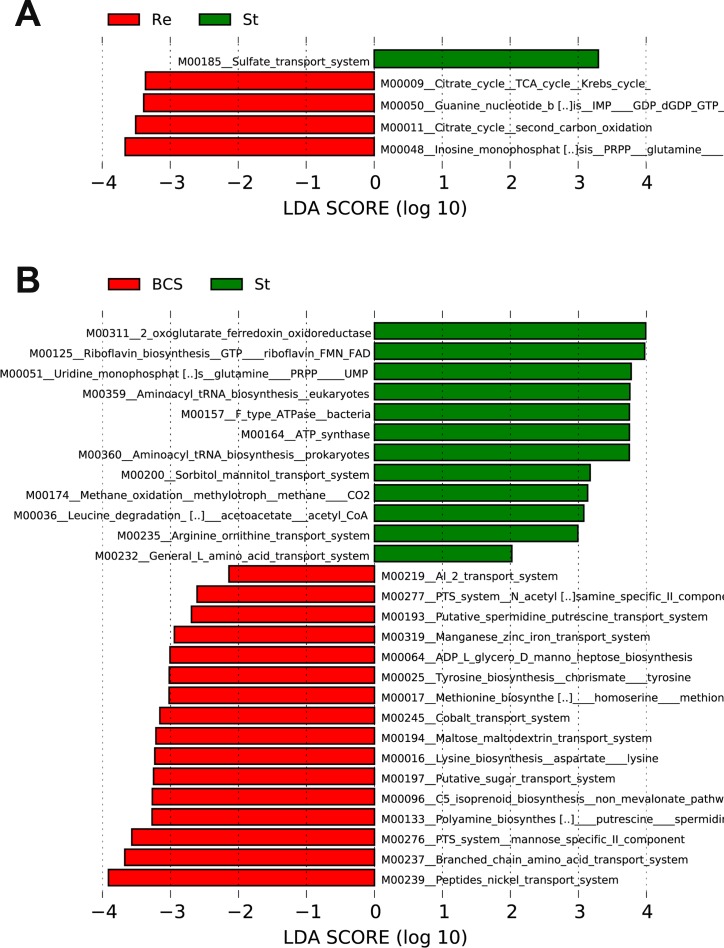
Identification of specific microbial functions associated with B-CS stability (**A**) Differences in gut microbial functions between BCS recurrent group and B-CS stable group based on the LDA scores (log_10_). (**B**) Differences in gut microbial functions between B-CS group and B-CS stable group based on the LDA scores (log_10_). LDA, linear discriminant analysis.

Moreover, to assess the potential microbial functions associated with B-CS stability, we used PICRUSt pipeline [22] to predict microbial functions. The LDA scores (log_10_) indicated that the sulphate transport system was enriched, whereas PRPP glutamine biosynthesis, the citrate cycle and guanine nucleotide biosynthesis were reduced, in stable group versus recurrent group (Figure 8A, Supplementary Data File 7). Moreover, 7 microbial functions, mainly ferredoxin oxidoreductase activity, riboflavin biosynthesis, PRPP glutamine biosynthesis and tRNA biosynthesis, were enriched in stable group compared with B-CS group, whereas 16 functions, mainly involving the peptide/nickel transport system, branched-chain amino acid transport system and arginine biosynthesis, were decreased (Figure 8B, Supplementary Data File 8). Notably, PRPP glutamine biosynthesis was reduced in stable group versus recurrent group but enriched in stable group versus B-CS group, demonstrating a unique microbial feature of B-CS stability.

## DISCUSSION

In this study, we identified gut microbial diversity and species richness in B-CS patients versus LC patients. Moreover, the bacterial community of B-CS patients was clustered with that of controls but separated from that of LC patients. Shared OTU analysis demonstrated that OTUs 421, 502 (*Clostridium IV*) and 141 (*Megasphaera*) were unique to B-CS. The abundances of genera *Actinomyces*, *Rothia* and *Atopobium* were decreased in B-CS group versus LC group, whereas nine genera, mainly including *Bacteroides* and *Megamonas,* were enriched. Notably, the genus *Megamonas* could distinguish B-CS patients from LC patients (AUC: 0.7904). The L-amino acid transport system was decreased in B-CS patients versus LC patients and controls. In addition, a unique bacterial community (genus *Clostridium XI*) and microbial function (PRPP glutamine biosynthesis) associated with stability were also identified. To the best of our knowledge, this is the first report of the gut microbial characteristics of B-CS patients determined via MiSeq sequencing of a large cohort.

Gut microbial populations could serve as biomarkers of specific diseases. Qin *et al.* [21] previously analysed gut microbial alterations in LC and established an accurate patient discrimination index on the basis of 15 microbial biomarkers. In patients with primary sclerosing cholangitis (PSC), the *Veillonella* genus was shown to yield an AUC of 0.64 for discriminating PSC patients from healthy controls, whereas a combination of PSC-associated genera yielded an AUC of 0.78, suggesting a potential microbial biomarker for PSC [24]. In addition, Yu *et al.* [25]. identified four microbial gene markers for distinguishing colorectal cancer (CRC) from control metagenomes, with AUCs of 0.72 and 0.77 in the discovery cohort and validation cohort, respectively. Quantitative PCR analysis of these genes accurately classified the CRC patients in the independent Chinese cohort with an AUC of 0.84, highlighting the potential use of faecal metagenomic biomarkers for early CRC diagnosis. In our study, the genus *Megamonas* could distinguish B-CS from LC patients (AUC: 0.7904), and microbial function (L-amino acid transport system) was decreased in B-CS patients versus LC patients and controls. These results suggested that specific gut microbiota alterations might represent non-invasive biomarkers for B-CS diagnosis.

Most LC patients have gut bacterial overgrowth [26]; thus, they exhibit not only taxonomic differences in microbial communities but also increased intestinal bacterial burdens compared with individuals without cirrhosis. Gut microbiota has been reported to display increases in potentially pathogenic bacteria, including *Enterobacteriaceae*, *Veillonellaceae* and *Streptococcaceae*, and reductions in beneficial populations, such as *Lachnospiraceae* [16]. In this study, the gut microbiota of B-CS patients also exhibited significant dysbiosis. However, in contrast with LC patients, the genera *Actinomyces*, *Rothia* and *Atopobium* were decreased, most of which were anaerobic, and nine genera, mainly including *Bacteroides* and *Megamonas,* were enriched in B-CS patients, and these genera were significantly correlated with endotoxin and systemic inflammatory cytokines. Notably, the genus *Megamonas* could distinguish B-CS patients from LC patients (AUC: 0.7904). Microbial function prediction demonstrated that the L-amino acid transport system was dramatically decreased in B-CS patients versus LC patients and healthy controls. As a common indicator of organ metabolism and nutrition, this decrease probably contributed to the difference in these patients, especially in the gut and liver. Interestingly, shared OTU analysis demonstrated that OTUs 421 and 502 (*Clostridium IV*) were markedly increased, whereas taxonomic analysis revealed that *Bacteroides*, *Megamonas*, *Oscillibacter* and *Alistipes*, but not *Clostridium IV*, were enriched in B-CS patients versus healthy controls and LC patients. These results appear to be contradictory but are actually attributed to the notion that the same genus may be derived from different OTUs. The enrichment of *Bacteroides* in B-CS patients would result in increased lipopolysaccharide (LPS) secretion, which might elicit strong immune responses and activate the NF-kB pathway, leading to proinflammatory cytokine production (TNF-a, IL-6 and IL-1) and liver inflammatory and oxidative damage [27]. Thus, these results indicated that the unique gut microbiota characteristics of B-CS patients played pivotal roles in disease pathogenesis, in contrast with those of LC patients.

Our previous studies have revealed that formation of intra- and extra-hepatic collaterals during the chronic course of B-CS leads to improved liver function and the relief of intestinal congestion; thus, collateral circulation is of great clinical importance to B-CS treatment strategies [4, 28]. Considering the compensatory environment caused by collateral circulation, we classified B-CS patients into six pathophysiological subtypes. These patients were treated using different strategies, with satisfactory results. Moreover, liver function and intestinal status are closely associated with the gut microbiota through liver-gut circulation and the gut microbiota-liver axis [19]. The improvement of liver function may result in the amelioration of gut barrier function and promote gut microbial restoration, which may further benefit the injured liver through positive feedback of the “gut-liver axis.” [15] In this study, gut microbial diversity and species richness were significantly decreased in LC patients versus healthy controls but markedly increased in B-CS versus LC patients. Importantly, PCA revealed that the bacterial community of B-CS patients was clustered with that of healthy controls but was clearly separated from that of LC patients, suggesting obvious dysbiosis of the gut microbiota in LC and its significant restoration in B-CS versus LC patients. Taken together, these results demonstrated that collateral circulation was greatly contributed to gut microbial restoration in B-CS patients through the improvement of liver function and remission of intestinal congestion.

Importantly, shared OTU analysis indicated that OTUs 27 (*Clostridium XI*), 137 (*Clostridium XIVb*) and 40 (*Bacteroides*) were associated with B-CS stability. Furthermore, the genus *Clostridium XI* was enriched in stable patients versus recurrent group and B-CS group, consistent with the OTU analysis results. These findings indicated that increased *Clostridium XI* greatly contributed to stability after B-CS treatment. *Clostridium XI* are short-chain fatty acid (SCFA)-producing bacteria, including *Clostridium difficile*, *Clostridium litorale*, and *Clostridium lituseburense,* which have been reported to be increased in irritable bowel syndrome patients [29]. SCFAs beneficially influence the development of inflammation-related pathologies, and their production can be influenced by the diet [30–33]. Moreover, they affect both cell morphology and function, possess anti-oxidative, anti-carcinogenic and anti-inflammatory properties and play essential roles in maintaining gastrointestinal and immune homeostasis [34–36]. Thus, these functions of *Clostridium XI*-produced SCFAs may be involved in gut microbial homeostasis, thereby maintaining stability post-B-CS treatment.

Another important finding of this study was the association of a decrease in LPS-producing bacteria with the maintenance of stability post-B-CS treatment. Our results indicated that the families *Enterobacteriaceae* and *Enterococcaceae* and the genera *Escherichia/Shigella* were significantly decreased in stable group versus B-CS group. As common Gram-negative bacteria, these bacterial populations may produce LPS to elicit strong immune responses and initiate various pathophysiological cascades, whereas LPS act as prototypical endotoxins and promote pro-inflammatory cytokine, nitric oxide, and eicosanoid secretion [37]. The enrichment of SCFA-producing bacteria and decrease in LPS-producing bacteria collectively result in the maintenance of stability post-B-CS treatment and reduced B-CS recurrence. Therefore, gut microbiota-targeted therapy or faecal microbiota transplantation of specific bacteria may be a novel strategy for preventing or reducing recurrence post-B-CS treatment.

In conclusion, gut microbial diversity was increased in B-CS patients versus LC patients. Collateral circulation greatly contributed to gut microbial restoration in B-CS patients through improved liver function and the remission of intestinal congestion. Genus *Megamonas* could distinguish B-CS patients from LC patients, suggesting its potential as a non-invasive biomarker for B-CS. The enrichment of SCFA-producing bacteria and reduction in LPS-producing bacteria resulted in the maintenance of stability post-B-CS treatment and reduced B-CS recurrence. Therefore, specific alterations associated with prognosis were detected in gut microbiota of B-CS patients. Correction of gut microbial alterations may warrant consideration as a potential strategy for B-CS prevention and treatment.

## MATERIALS AND METHODS

### Participant information

This study was designed and conducted according to the ethical guidelines of the 1975 Declaration of Helsinki and approved by the Institutional Review Board of the First Affiliated Hospital of Zhengzhou University (reference number 2010–05). Written informed consent were obtained from all participants, and patient information were collected (Supplementary Table 1). This study complied with the principle of PRoBE (prospective specimen collection and retrospective blinded evaluation) design [38].

B-CS diagnosis is based on demonstration of hepatic venous outflow tract obstruction [1]. Doppler ultrasound, triphasic CT imaging or MRI was used to identify these diagnostic characteristics. LC was diagnosed by the comprehensive integration of imaging findings, clinical symptoms and physical signs, laboratory test results and medical history. All B-CS patients did not suffer from transjugular intrahepatic portosystemic shunt (TIPS) treatment. The treatment strategy of B-CS patients was consistent with international guidelines. Stable B-CS patients (Stability) were defined as those without recurrence during 2 years post-B-CS treatment. Alternatively, recurrent B-CS patients (Recurrence) were defined as those with recurrence during 2 years post-treatment. Stool samples were collected from the stable and recurrent B-CS patients after they had not shown any clinical symptoms (stabilization) after at least 6 months.

Patients with the following criteria were excluded: the presence of (a) severe complications; and/or (b) another disease, such as cancer, intestinal disease, hypertension or metabolic disease. Additional inclusion criteria were as follows: age of 30-70 years, normal renal function, and body mass index (BMI) >20.

Correspondingly, healthy volunteers for annual physical examination were enrolled as healthy controls (H). The inclusion criteria for the healthy volunteers were as previously described [21]. The exclusion criteria for the healthy volunteers included hypertension, diabetes, obesity, metabolic syndrome, inflammatory bowel disease (IBD), non-alcoholic fatty liver disease, coeliac disease and LC. All participants who received antibiotics and/or probiotics within 8 weeks before enrolment were also excluded.

### Sample collection and DNA extraction

Each subject before concomitant medication provided a fresh stool sample, which was delivered immediately from the hospital to the laboratory in a bag on ice. At the laboratory, the stool sample was immediately stored at –80°C. DNA extraction from a frozen aliquot of each stool sample was conducted as previously described [16, 18].

### PCR amplification and MiSeq sequencing

The bacterial DNA extracted from the stool samples was amplified using a set of primers targeting the hypervariable V3-V5 region (338F/806R) of the 16S rDNA gene (forward and reverse primers: 5′-ACTCCTACGGGAGGCAGCA-3′ and 5′-GGACTACHVGGGTWTCTAAT-3′, respectively). A barcode and adapter were incorporated between the adapter and forward primer. PCR amplification was performed in a 20 μl reaction mixture as previously described [18, 21]. DNA libraries were constructed according to the manufacturer’s instructions, and sequencing was performed on an Illumina MiSeq platform at Majorbio Bio-Pharm Technology Co., Ltd. (Shanghai, China).

### Sequence assembly process

Paired V4-16S rDNA sequences were trimmed to 200 bp and merged into a single sequence using FLASH v1.2.10 software [39] with the default parameters. Chimeric sequences were detected and removed with UCHIME version 4.2.40. [40] The 16S “golden standard” database was used as a reference (Broad Institute; version microbiome util-r20110519; http://drive5.com/uchime/gold.fa) for OTUs matching.

### OTU clustering and taxonomy annotation

Equal numbers of assembled reads (10,000 reads) were randomly extracted from all samples, and then the corresponding OTUs were binned using UPARSE pipeline [41] as follows: (a) abundant sequences and singletons were removed; (b) unique sequences were binned into OTUs with the command “usearch-cluster_otus”; and (c) randomly chosen sequences were aligned against OTU sequences with the command “usearch-usearch_global-id 0.97”, the identity threshold was set to 0.97, and an OTU table was then created. All selected sequences were annotated using RDP classifier version 2.6 [42] with a confidence level of 0.5, as recommended by the developer (http://rdp.cme.msu.edu/classifier/class_help.jsp#conf).

### Bacterial diversity and bacterial community analysis

Bacterial diversity was assessed by sampling-based analysis of OTUs and presented as a rarefaction curve. Bacterial richness and diversity indexes across the samples were calculated as Shannon’s index, the Chao 1 index and ICEs, which were estimated at a distance of 3% using the R package “vegan.” [43] PCA was performed based on OTU abundance and distribution using R software (http://www.r-project.org/) to analyse bacterial communities. Weighted and unweighted UniFrac distances were calculated with phyloseq package. [44] Then, bacterial communities were compared at the phylum, class, order, family and genus levels.

The linear discriminant analysis (LDA) effect size (LEfSe) method was used to identify the greatest differences among specific characteristics of intestinal microbiotas [45] (http://huttenhower.sph.harvard.edu/lefse/). With a normalized relative abundance matrix, the LEfSe method uses the Kruskal-Wallis rank sum test to detect characteristics with markedly different abundances among assigned bacterial taxa, as well as LDA to assess the effect size of each characteristic [46].

### Functional annotation of gut microbial 16S rDNA gene

To predict the functional profiles of gut microbial communities based on 16S rDNA gene sequences, we utilized PICRUSt version 1.0.0 [22] and human version 0.99 [23] to establish KEGG orthologies (KOs) and KEGG pathway/module profiles.

### Statistical analysis

Continuous data are presented as either the mean ± standard deviation (SD) for normally distributed variables or the median (interquartile range) for non-normally distributed variables, and they were analysed between groups using the Wilcoxon rank-sum test. Categorical variables are expressed as group percentages and were compared among samples using either Pearson’s χ^2^ or Fisher’s exact test. All statistical tests were 2-sided, with a significance level of *P* < 0.05. Receiver operating characteristic (ROC) curves were generated and areas under the curve (AUCs) were calculated to evaluate the ROC effects. Statistical analyses were conducted using SPSS version 19.0 for Windows (SPSS Inc., Chicago, IL, USA).

## SUPPLEMENTARY MATERIALS FIGURES AND TABLE





















## Abbreviations

B-CS: Budd–Chiari syndrome
IVC: inferior vena cava
HCC: hepatocellular carcinoma
LC: liver cirrhosis
BMI: body mass index
IBD: inflammatory bowel disease
OUT: Operational Taxonomic Unit
ICEs: incidence-based Coverage Estimators
PCA: principal component analysis
LDA: linear discriminant analysis
ROC: receiver operating characteristic
AUCs: areas under the curve
PSC: primary sclerosing cholangitis
CRC: colorectal cancer
LPS: lipopolysaccharide
SCFA: short-chain fatty acid
