# Supplementary material for: Specific alterations in gut microbiota are associated with prognosis of Budd–Chiari syndrome
Source: Oncotarget. 2017 Dec 14;9(3):3303–20. doi: 10.18632/oncotarget.23234 (PMC5790465; doi:10.18632/oncotarget.23234)
Supplement: Supplementary file 1 [file oncotarget-09-3303-s001.pdf]

Specific alterations in gut microbiota are associated with prognosis of Budd–Chiari syndrome

SUPPLEMENTARY MATERIALS

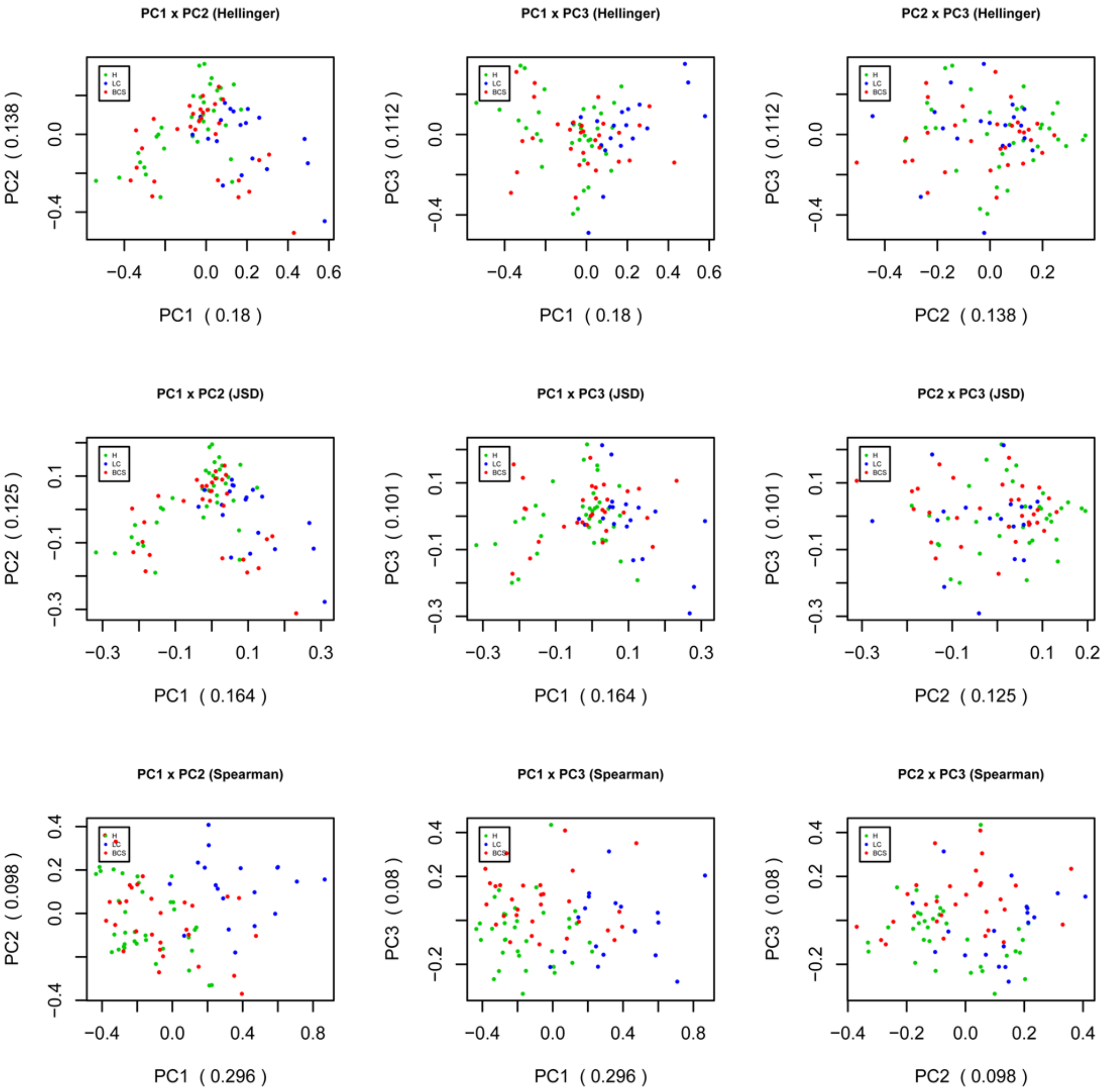

Supplementary Figure 1: Bacterial community distribution among healthy controls, liver cirrhosis patients and BCS patients using the different distances analysis. Three different distances analysis (Hellinger, JSD and Spearman analysis) were used to describe gut microbial distribution among healthy controls, liver cirrhosis patients and BCS patients. The results indicated that bacterial community in LC patients were clearly separated from healthy controls, but B-CS patients presented an obvious restoration of gut microbiota versus LC patients. H, healthy controls; LC, liver cirrhosis; BCS, Budd-Chiari syndrome.

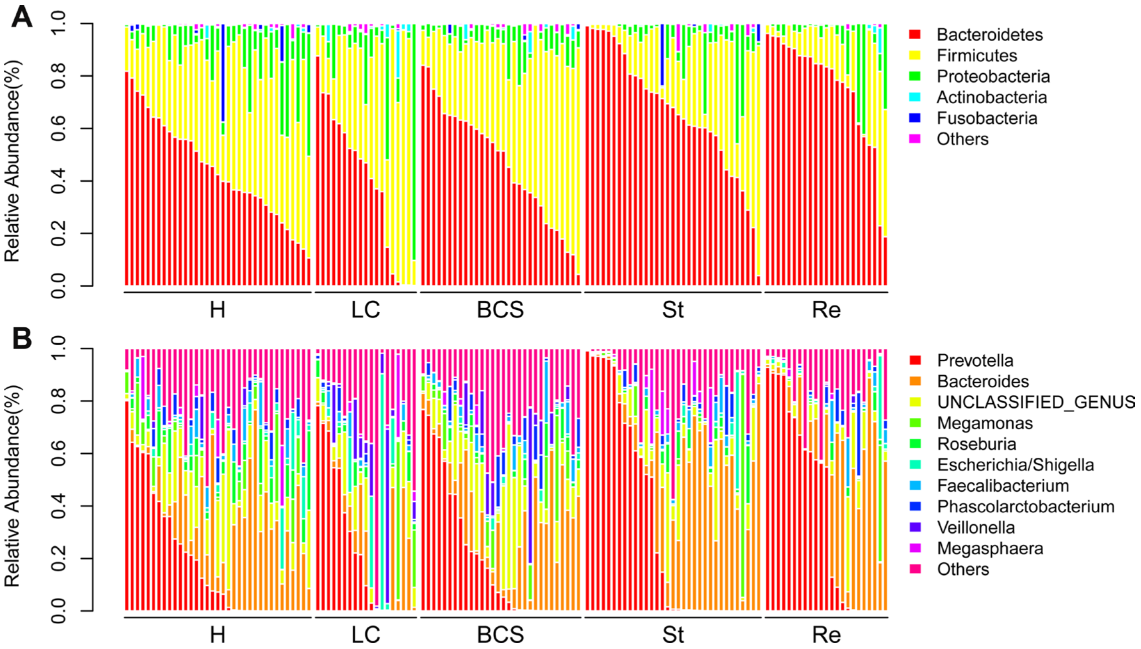

Supplementary Figure 2: Gut microbial abundance and composition at the phylum and genus levels for each sample among healthy controls, LC patients, BCS patients, stability patients and recurrence patients. (**A**) Bacterial abundance and composition of the top 5 phylum for each sample among the five groups. (**B**) Bacterial abundance and composition of the top 10 genus for each sample among the five groups. H, healthy controls; LC, liver cirrhosis; B-CS, Budd-Chiari syndrome; St, Stability group; Re, Recurrence group.

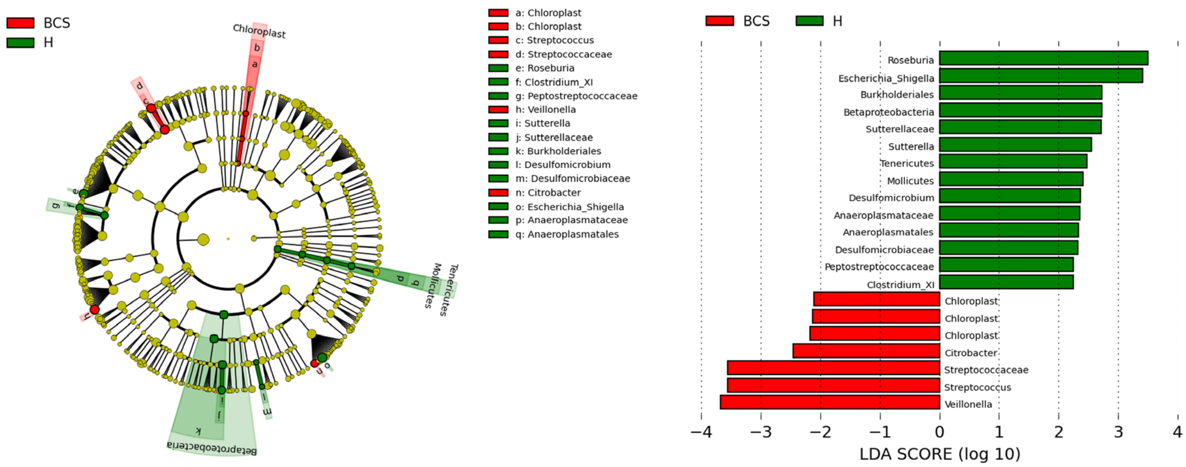

Supplementary Figure 3: Identification of the specific bacterial taxa between the BCS patients and healthy controls. (left) Phylogenetic profiles of the specific bacterial taxa and the predominant bacteria in the BCS patients versus healthy controls using the LEfSe method. (right) The greatest differences in taxa between the BCS patients and healthy controls were shown by LDA score ($\log_{10}$). H, healthy controls; B-CS, Budd-Chiari syndrome;

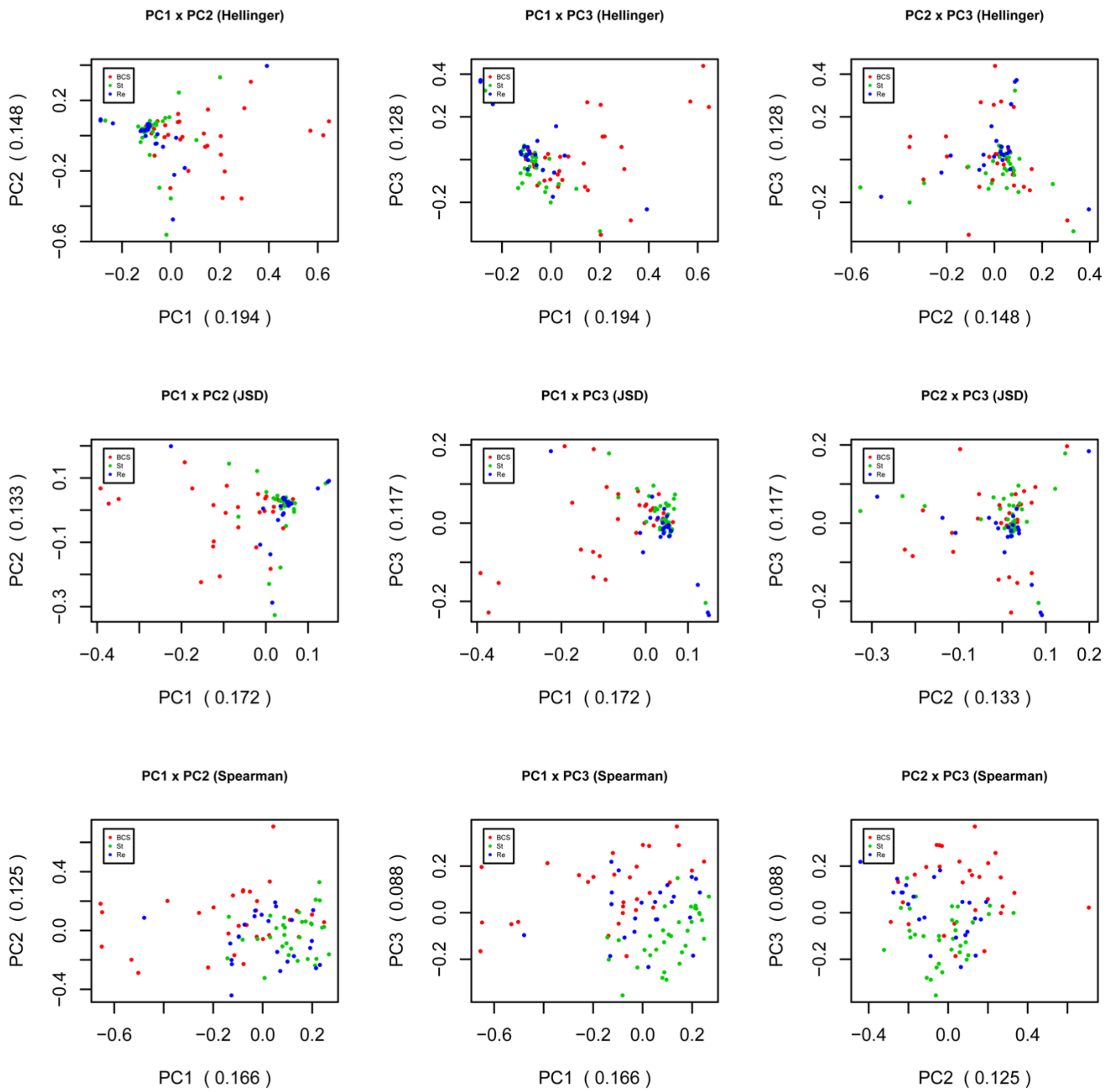

Supplementary Figure 4: Bacterial community distribution among BCS patients, stability patients and recurrence patients using the different distances analysis. Three different distances analysis (Hellinger, JSD and Spearman analysis) were used to describe gut microbial distribution among BCS patients, Stability patients and Recurrence patients. Gut microbial community in B-CS patients was dramatically separated from Stability and Recurrence groups, while no significant separation was observed between Stability and Recurrence groups based on the different distances analysis (Hellinger, JSD and Spearman analysis). B-CS, Budd-Chiari syndrome; St, Stability group; Re, Recurrence group.

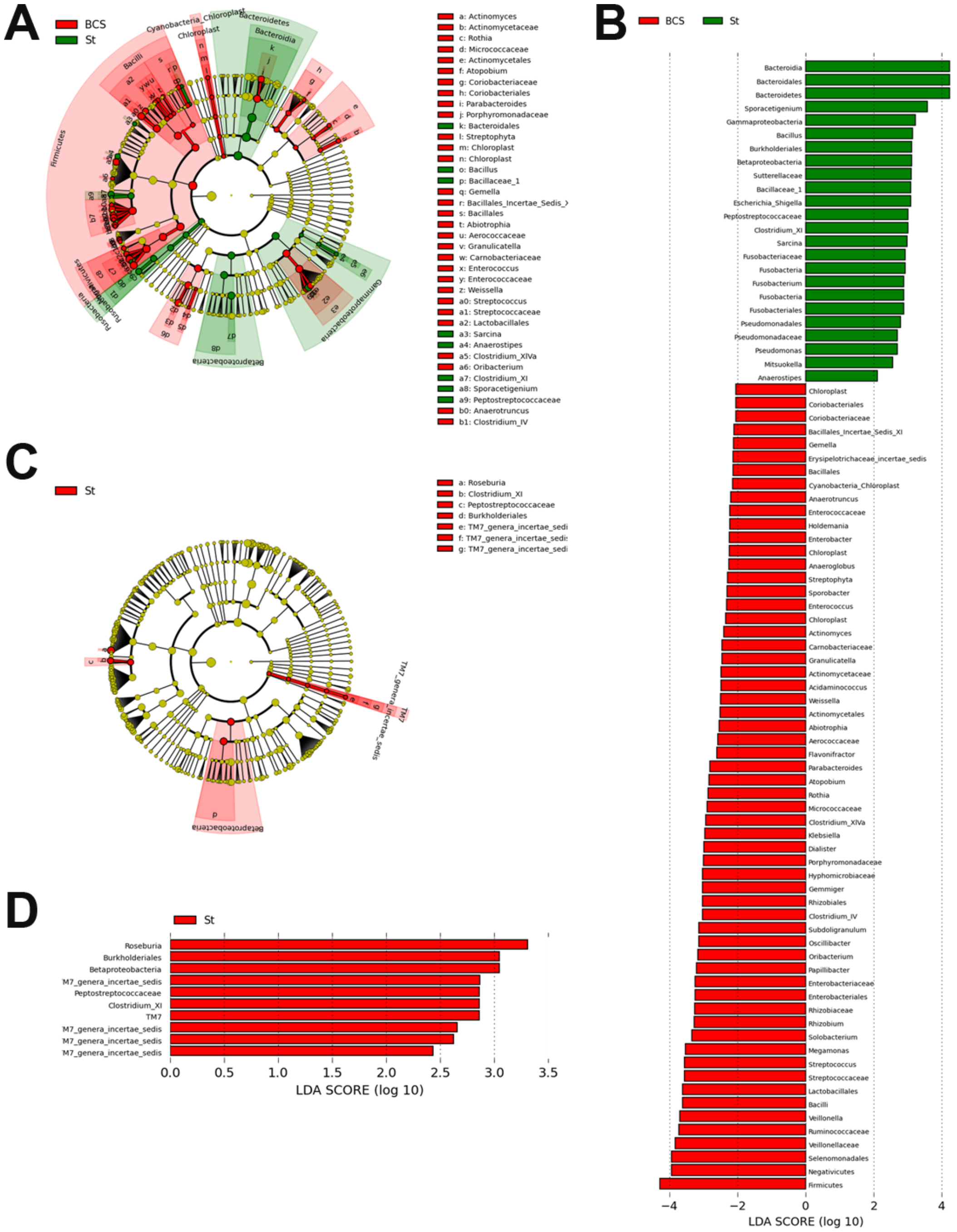

Supplementary Figure 5: Identification of the specific bacterial taxa between the BCS patients and stability group as well as the stability group and the recurrence group. (**A**) Phylogenetic profiles of the specific bacterial taxa and the predominant bacteria between BCS patients and Stability patients using the LEfSe method. (**B**) The greatest differences in taxa between BCS patients and Stability patients were shown by LDA score ($\log_{10}$). (**C**) Phylogenetic profiles of the specific bacterial taxa and the predominant bacteria between Stability patients and Recurrence patients using the LEfSe method. (**D**) The greatest differences in taxa between Stability patients and Recurrence patients were shown by LDA score ($\log_{10}$). B-CS, Budd-Chiari syndrome; St, Stability group; Re, Recurrence group.

Supplementary Table 1: Informed consent form and information collection in the study (Translated from Chinese). See Supplementary_Table_1

Supplementary Data File 1: The detailed clinical information of the enrolled 144 subjects. See Supplementary_Data_File_1

Supplementary Data File 2: Taxonomic annotations were performed for all qualified operational taxonomic units (OTUs). See Supplementary_Data_File_2

Supplementary Data File 3: The distribution and abundance of the 560 qualified OTUs among the different samples. See Supplementary_Data_File_3

Supplementary Data File 4: The diversity and species richness index of gut microbiota among the different samples. See Supplementary_Data_File_4

Supplementary Data File 5: The abundance and difference of gut microbial gene function between the B-CS patients and LC patients. See Supplementary_Data_File_5

Supplementary Data File 6: The abundance and difference of gut microbial gene function between the B-CS patients and healthy controls. See Supplementary_Data_File_6

Supplementary Data File 7: The abundance and difference of gut microbial gene function between the stable group and the recurrent group. See Supplementary_Data_File_7

Supplementary Data File 8: The abundance and difference of gut microbial gene function between the stable group and the B-CS group. See Supplementary_Data_File_8
