# Supplementary material for: Specific alterations in gut microbiota are associated with prognosis of Budd–Chiari syndrome
Source: Oncotarget. 2017 Dec 14;9(3):3303–20. doi: 10.18632/oncotarget.23234 (PMC5790465; doi:10.18632/oncotarget.23234)
Supplement: Supplementary file 2 [file oncotarget-09-3303-s002.pdf]

Informed consent form and information collection

(Translated from Chinese)

We are from Key Laboratory of Combined Multi-organ Transplantation, Ministry of Public Health; Department of Hepatobiliary and Pancreatic Surgery, First Affiliated Hospital, School of Medicine, Zhejiang University. We will free of charge help you monitor your gut microbial community, thereby analyzing whether gut microbiota is dysbiosis and the degree of imbalance. These results will provide auxiliary data for clinical diagnosis and treatment. Now, you just provide stool and urine according to our instruction. The whole process keeps free of charge. These results will be used for scientific research. Thank you for your corporation.

Number:

Diagnosis:

Patient Sign：

Date:

Patient information collection

Name	Gender	Birth date	Height (cm)	Weight (kg)	BMI	Tel
Floor ward	Admission number	Dietary habit (vegetarian diet/meat/Mixture)	Antibiotics use within 2 months	Yoghourt and probiotics	Previously critical Illness	Long-term drug use
Drinking	Alcohol type	Drinking quantity	Time of Du ration	Whether or not abstinence	HBV	Etiology
